# Plasma Exosome-derived MicroRNAs as Novel Biomarkers of Traumatic Brain Injury in Rats

**DOI:** 10.7150/ijms.39667

**Published:** 2020-02-04

**Authors:** Pengcheng Wang, Haoli Ma, Yuxian Zhang, Rong Zeng, Jiangtao Yu, Ruining Liu, Xiaoqing Jin, Yan Zhao

**Affiliations:** 1 Emergency Center, Zhongnan Hospital of Wuhan University, Wuhan 430071, China.; 2 Hubei Clinical Research Center for Emergency and Resuscitation, Zhongnan Hospital of Wuhan University, Wuhan 430071, China.

**Keywords:** Traumatic brain injury, exosomes, microRNA, plasma, biomarker, rat.

## Abstract

Traumatic brain injury (TBI) is a widespread central nervous system (CNS) condition and a leading cause of death, disability, and long-term disability including seizures and emotional and behavioral issues. To date, applicable diagnostic biomarkers have not been elucidated. MicroRNAs (miRNAs) are enriched and stable in exosomes in plasma. Therefore, we speculated that miRNAs in plasma exosomes might serve as novel biomarkers for TBI diagnosis and are also involved in the pathogenesis of TBI. In this study, we first isolated exosomes from peripheral blood plasma in rats with TBI and then investigated the alterations in miRNA expression in exosomes by high-throughput RNA sequencing. As a result, we identified 50 significantly differentially expressed miRNAs, including 31 upregulated and 19 downregulated miRNAs. Then, gene ontology (GO) and Kyoto Encyclopedia of Genes and Genomes (KEGG) pathway analysis revealed that the most highly correlated pathways that were identified were the MAPK signaling pathway, regulation of actin cytoskeleton, Rap1 signaling pathway and Ras signaling pathway. This study provides novel perspectives on miRNAs in peripheral blood plasma exosomes, which not only could be used as biomarkers of TBI diagnosis but could also be manipulated as therapeutic targets of TBI.

## Introduction

Due to the increasing incidence of traffic accidents and falls, an increasing number of people every year seek medical care for traumatic brain injury (TBI). Among these individuals, 75% or more are considered to have a mild or moderate TBI 1. Although severe TBI is one of the main causes of death or disability in patients, mild or moderate TBI is a leading cause of long-term disability including seizures and emotional and behavioural issues 2. The damage to the cranium and the intracranial contents caused by a TBI can be divided into the primary injury and delayed secondary injury, which is more profound and includes axonal shearing 3, neuroinflammation 4,5, neurochemical changes 6, 7, brain edema 8, vascular injury 9, cell apoptosis 10 and mitochondrial dysfunction 11.

Currently, strategies for diagnosing and treating TBI in patients are limited 12. Imaging tests (computed tomography, CT or magnetic resonance imaging, MRI) are generally applied to diagnose and classify TBI in clinical practice but are limited because of low sensitivity in patients with mild injuries 13, 14. Cerebrospinal fluid (CSF) or blood tests are useful in the assessment of the severity of the injury and subsequent recovery of TBI patients by detecting protein or miRNA components in the peripheral blood 14. For instance, protein tests that can detect cytokines, chemokines, tau, calcium-binding protein S100B, glial fibrillary acidic protein (GFAP), neuron-specific enolase (NSE), ubiquitin carboxy-terminal hydrolase L1 (UCH-L1), and myelin basic protein (MBP) are candidates for classification and prognostication of TBI patients 15. However, these tests have detection sensitivity problems due to the low concentration of the substances (fM-pM). MiRNAs, which are small (19-23 nt) endogenous RNA molecules, are post-transcriptional regulators of gene expression and protein synthesis, bind to target sites in the noncoding region of mRNA, are enriched and stable in exosomes and are involved in the onset of many diseases. Microarray and sequencing are the most often used methods for miRNA quantitation. We chose the latter method because sequencing yields more precise accuracy and the ability to investigate the novel structure of miRNA. Furthermore, microRNAs (miRNA) could be indicators of the pathology of TBI. Serum or plasma miRNAs have been reported as specific and sensitive biomarkers of many CNS conditions 16, 17, including TBI 18-21. For example, a subset of ten unique miRNAs was shown to have the ability to diagnose and distinguish between mild/moderate TBI and severe TBI 22, 23. However, one constraint is that miRNAs can easily dissolve or degrade in plasma samples.

Recently, several studies have suggested that extracellular vehicles (EVs) might play a vital role in TBI pathogenesis 24. Exosomes are EVs with diameters that typically range from 40-150 nm. These particles have a phospholipid bilayer that carries proteins, metabolites, lipids, mRNAs, and miRNAs that are released from cells and could be an important medium for cell-cell or cell-internal environment communication 25. After trauma, brain cell-derived exosomes containing intracranial substances cross the blood-brain barrier (BBB) and are transferred to the peripheral blood. Therefore, they might serve as diagnostic and prognostic biomarkers. MiRNAs have been shown to be stable in exosomes and EVs 25. Alteration of miRNA expression has also shown to occur with disease onset and progression. Therefore, we conjectured that miRNAs in exosomes and EVs in the peripheral blood plasma after TBI might play a vital role in regulating gene expression, protein synthesis and specific signaling pathways, which could constitute a good biomarker for diagnosis.

The present study aimed to identify vital miRNAs, provide novel targets for further study, and evaluate the alterations in miRNA expression in exosomes in the plasma after TBI by high-throughput RNA sequencing. Bioinformatics analysis was used to predict the function of differentially expressed miRNAs by gene ontology (GO) and Kyoto Encyclopedia of Gene and Genomes (KEGG) pathway analysis. Pathway relationship networks was constructed based on differentially expressed miRNAs to explore the interactions among them. In addition, 11 miRNAs were randomly selected to confirm the reliability of the microRNA sequencing results by quantitative reverse transcription-polymerase chain reaction (qRT-PCR). This study revealed several novel potential roles of these differentially expressed miRNAs in plasma exosomes from the peripheral blood after TBI.

## Materials and Methods

### Experimental Animals

Experiments were performed using male adult SPF-grade SD rats (12--16 weeks old and 300-400 g, purchased from the Animal Center of Wuhan University) that were acclimated at least one week before surgery. All animal experiment protocols were approved by the Animal Experiment Center and Ethics Committee of Zhongnan Hospital of Wuhan University and followed the National Institutes of Health Guide for the Care and Use of Laboratory Animals.

### TBI model and experimental groups

In this study, we used the weight-drop (WD) method as described in previous studies to induce moderate TBI in rats 26, 27. Briefly, the rats were intraperitoneally anaesthetized with 1% pentobarbital (30 mg/kg). After anaesthesia, the rats were fixed, the hair was shaved, and the skin was sterilized and cut. The bregma was exposed, and a 5-mm-diameter hole (2.0 mm posterior from the bregma and 2.0 mm right of the sagittal suture) was drilled into the skull; then, the dura mater was exposed. A 40 g -weight was used for a vertical strike at 25 cm, resulting in moderate TBI. The sham group received surgery but did not undergo the WD.

After 24 hours, all rats were anaesthetized and peripheral blood was collected in a tube containing EDTA as an anticoagulant. The samples were stored at 4°C for a short time and immediately centrifuged at 4°C and 3000 rpm for 10 min. The supernatant was carefully collected and transferred to a new tube without disturbing the intermediate buffy coat layer, and then the sample was passed through a 0.8 μm filter (SLAA033SB, Millex, Millipore) to remove additional cellular fragments and cell debris. Then the samples were collected for further steps. In total, 14 samples (seven from the TBI group and seven from the sham group) were used to isolate exosomes and extract miRNAs. Among them, six samples (three from the sham group and three from the TBI group) were used for miRNA sequencing, and eight samples (four from the sham group and four from the TBI group) were chosen for qRT-PCR validation.

### Isolation and detection of exosomes

Exosomes derived from plasma were isolated from the plasma using an exoEasy Maxi Kit (Qiagen, 76064) following the manufacturer's instructions. Exosomes were observed under a transmission electron microscope (TEM; FEI Tecnai G2 spirit, The Czech Republic), stained with phosphotungstic acid and detected at a voltage of 80kV. A nanoparticle tracking analyser (NTA) (ZetaView) was used to determine the size distribution of the plasma-derived exosomes.

The proteins were extracted from plasma exosomes of both groups. Western blotting was performed to detect typical exosomal markers using WES, an automated capillary-based size sorting system (ProteinSimple, San Jose CA). The data were analysed using Compass software (ProteinSimple, San Jose CA). The primary antibodies used included CD63 (SBI, EXOAB-CD63A-1, 1:50), HSP70 (SBI, EXOAB-Hsp70A-1, 1:50), and Transferrin (Abcam, ab82411, 1:50), which was used as a reference control.

### microRNA sequencing and subsequent bioinformatics analyses

The miRNA was isolated from exosome samples using an exoRNeasy Serum/Plasma Maxi Kit (Qiagen, 77064) following the manufacturer's instructions. The sequence library was determined by an Agilent 2100 bioanalyser. Sequencing and analysis of small RNAs were conducted by OE biotech Co. Ltd. (Shanghai, China). The basic reads were converted into raw data by base calling and were obtained from an Illumine Hiseq4000 sequencer. Low-quality reads were filtered, and reads with 5' primer contaminants or poly (A) sequences were removed. Reads without a 3'adapter and insert tag and reads shorter than 15 nt or longer than 41 nt were filtered from the raw data, and the clean reads were obtained.

For the primary analysis, the length distribution of the clean sequences in the reference genome was determined. Non-coding RNAs were annotated as rRNAs, tRNAs, small nuclear RNAs (snRNAs), and small nucleolar RNAs (snoRNAs). These RNAs were aligned and then subjected to BLAST 28 searches against the Rfam v.10.1 (http://www.sanger.ac.uk/software/Rfam) 29, 30 and GenBank databases (http://www.ncbi.nlm.nih.gov/genbank/). Known miRNAs were identified by alignment against the miRBase v.21 database (http://www.mirbase.org/) 30, and the expression patterns of known miRNAs in different samples were analysed. Unannotated small RNAs were analysed by miRDeep2 31 to predict novel miRNAs. The corresponding miRNA star sequence was also identified based on the hairpin structure of the pre-miRNA and the miRbase database.

Differentially expressed miRNAs were identified with a threshold *p*-value < 0.05. The *p*-value was calculated with the DEG algorithm in the R package. The targets of differentially expressed miRNAs were predicted using Targetscan, miRDB, and Diana software, and the interaction was selected as the final decision.

GO enrichment (http://www.geneontology.org) and KEGG (http://www.genome.jp/kegg) pathway enrichment analyses of differentially expressed miRNA-target -genes were performed using R based on the hypergeometric distribution.

### qRT-PCR analysis

To verify the accuracy of the miRNA sequencing data, qRT-PCR was performed as a validation method. The target RNA and housekeeping genes of each sample were subjected to RT-PCR reactions on a CFX Connect Real-Time PCR System (Bio-Rad; CFX Maestro 1.0 software) with TB Green Premix Ex Taq II (Tli RNaseH Plus) (RR820A, TaKaRa, Japan).

RNA was reverse transcribed to synthesize cDNA using a Mir-X miRNA First Strand Synthesis Kit (638313, TaKaRa, Osaka, Japan); The PCR and premier design were performed according to the manufacturer's instructions. Briefly, RNA samples were mixed with mRQ buffer and mRQ Enzyme, preferably in a thermal cycler and incubated for 1 h at 37°C then the reaction was terminated at 85°C for 5min to inactivate the enzymes. When finished, a 9-fold volume of RNase-free water was added.

All procedures were performed as follows: 95°C, 30 sec; 45 PCR cycles (95°C, 5 sec; 60°C, 30 sec [plate read]); 95°C, 10 sec. The temperature was slowly increased from 65°C to 95°C to establish the melting curve of the PCR product (automatic instrument, ramp rate 0.05°C /s). The primers of the randomly selected miRNAs and the internal parameters of U6 are shown in Table 3.

### Statistical analysis

All statistical analyses were performed in SPSS 23.0. Data are expressed as the mean ± SD. Student's *t*-test was used to evaluate differences between the two groups. A *p*-value<0.05 was considered to indicate a significant difference.

## Results

### Detection of exosomes

**Figure 1:**
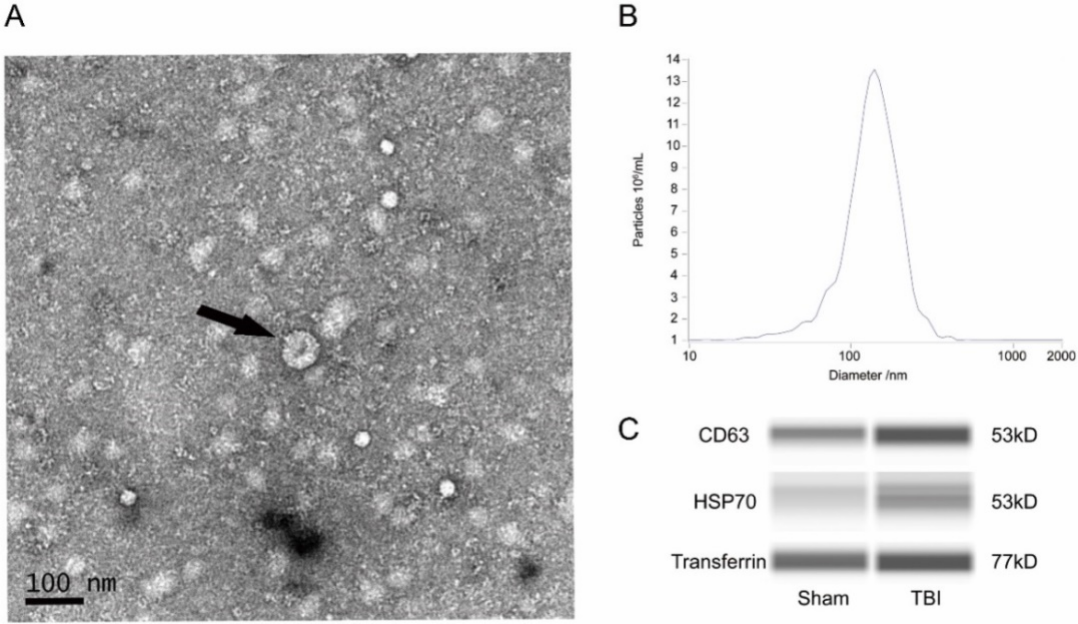
Detection of exosomes. (A) Representative transmission electron microscope (TEM) image of exosomes. TEM image of exosomes with negative staining to enhance the view of membrane structures (scale bar=100nm). Black arrow showed a typical exosome structure. (B) Representative nanoparticle tracking (NTA) analysis result of exosomes. The peak scale was measures as 140.3nm. (C) Representative western blot (WES) images of exosomes. Exosomes were probed for CD63, HSP70, and transferrin. CD, cluster of differentiation; HSP, heat shock protein.

Transmission electron microscope (TEM) analysis, nanoparticle tracking analysis (NTA) and western blot analysis were used to identify the exosomes that were obtained. In TEM images, the exosomes were irregular spheres ranging from 30-150 nm in diameter with a clearly defined and relatively intact membrane (Figure 1A). The NTA results showed a continuous range of exosomes, with a peak of 140.3nm (Figure 1B), suggesting a significant range of exosomes. The western blot analysis showed that the exosomes of both groups expressed the typical exosomal markers such as CD63 and HSP70 (Figure 1C).

### Differential expression of miRNAs in exosomes from the plasma after TBI

**Figure 2:**
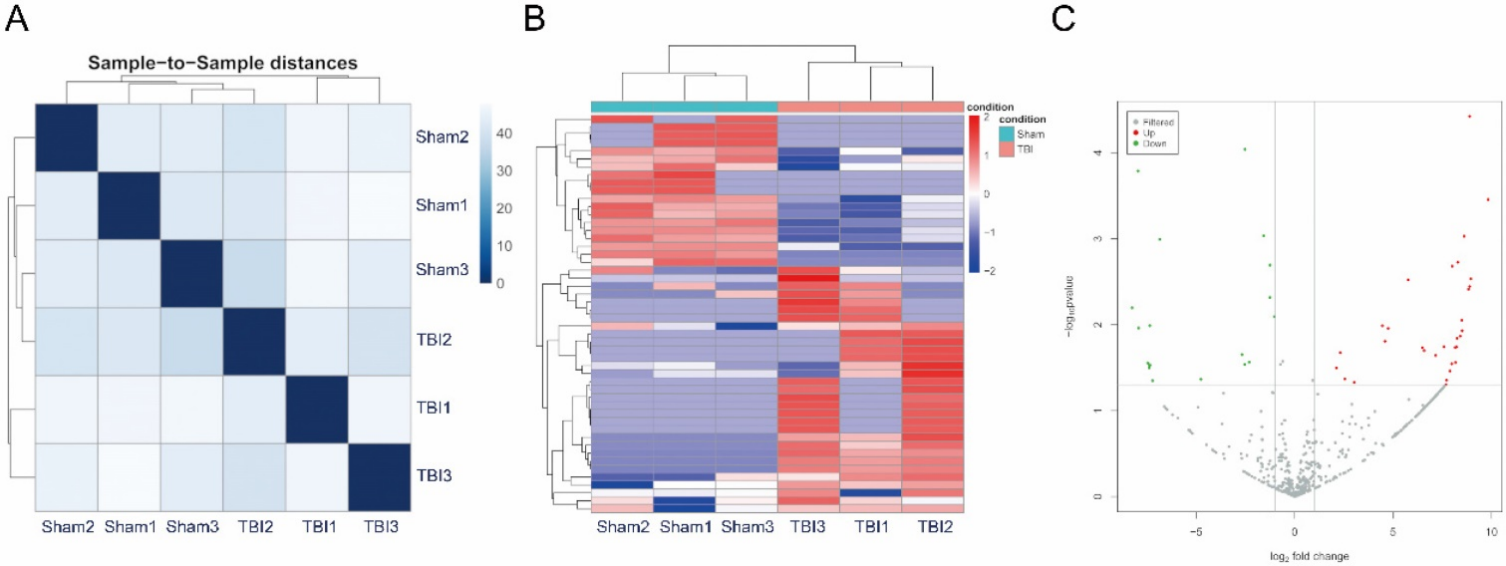
Correlation of samples, Differential expression of micro ribonucleic acids (miRNAs) in exosomes from plasma after traumatic brain injury (TBI). (A) The heatmap showed sample to sample distances, samples with high similarity were clustered together preferentially. (B) Heat plot of 50 differentially expressed miRNAs. Each column represents one sample; each row represents one probe set. The dendrogram on the top reveals the sample clustering; the dendrogram on the left reveals the gene clustering. (C) Volcano plot of expressions of miRNAs. Red spots were up-regulated miRNAs with significantly differential expressions, green spots were down-regulated, and gray spots were miRNAs with non-differential expressions. X-axis represented for log2 Fold Change; Y-axis represented for -log_10_ *P* value.

The miRNAs were sequenced in three TBI and three sham exosome samples from plasma. The miRNA-seq reads of each sample are shown in Table 1. The heatmap shows sample-to-sample distances, and samples with high similarity are preferentially clustered together (Figure 2A). We identified 50 significantly differentially expressed miRNAs that met the criteria of an expression fold change ≥ 2.0 and *p* value ≤ 0.05. Of these, 31 were upregulated and 19 were downregulated (Table 2). A heat map of 50 differentially expressed miRNAs was generated to illustrate the distinguishable miRNA expression profile of the samples (Figure 2B). A volcano plot was created according to the means of the expression values of the two groups of samples and the threshold of the expression fold change was 2.0 (Figure 2C).

### GO analysis of differentially expressed miRNA targeted genes

**Figure 3:**
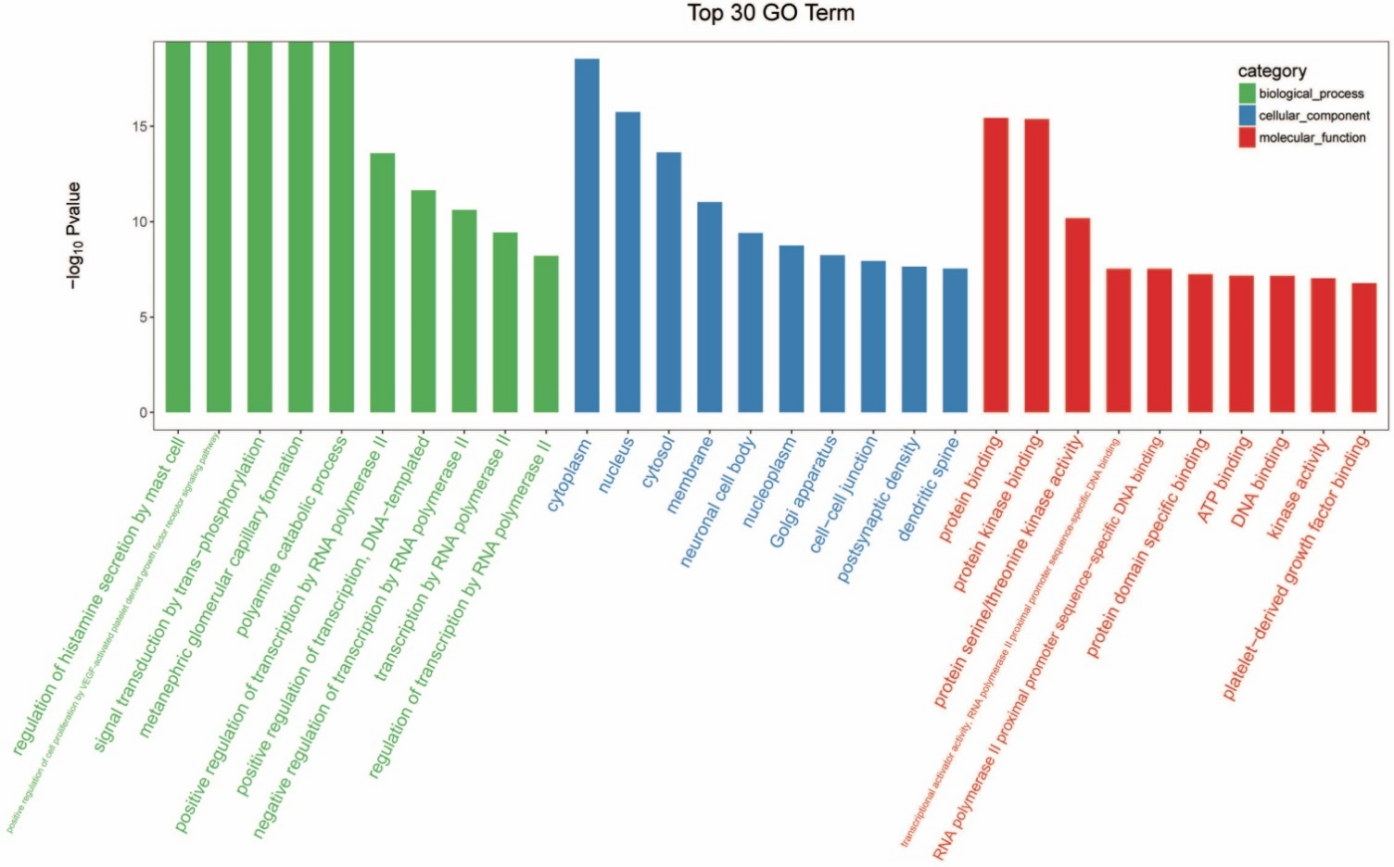
Gene ontology (GO) analysis of differentially expressed micro ribonucleic acids (miRNAs) with top 30 gene of -log_10_ *P* value in each GO terms.

The genes were subjected to GO function analysis to annotate and speculate the function of these miRNAs (Figure 3). GO analysis is divided into three parts: biological process (BP), cell component (CC), and molecular function (MF). GO analysis of the BP showed that differentially expressed miRNAs were significantly associated with the following terms: regulation of histamine secretion by mast cell; positive regulation of cell proliferation by VEGF-activated platelet derived growth factor receptor signaling pathway; signal transduction by trans-phosphorylation; metanephric glomerular capillary formation; polyamine catabolic process; positive regulation of transcription by RNA polymerase II; positive regulation of transcription, DNA-templated; negative regulation of transcription by RNA polymerase II; transcription by RNA polymerase II; and regulation of transcription by RNA polymerase II. The significant GO CC terms of differentially expressed miRNAs included the followings: cytoplasm, nucleus, cytosol, membrane, neuronal cell body, nucleoplasm, Golgi apparatus, cell-cell junction, postsynaptic density, and dendritic spine. For MF, differentially expressed miRNA were associated with the following terms: protein binding; protein kinase binding; protein serine/threonine kinase activity; transcriptional activator activity, RNA polymerase II proximal promoter sequence-specific DNA binding; RNA polymerase II proximal promoter sequence-specific DNA binding; protein domain specific binding; ATP binding; DNA binding; kinase activity; and platelet-derived growth factor binding.

### KEGG pathway analysis of differentially expressed miRNA targeted genes

**Figure 4:**
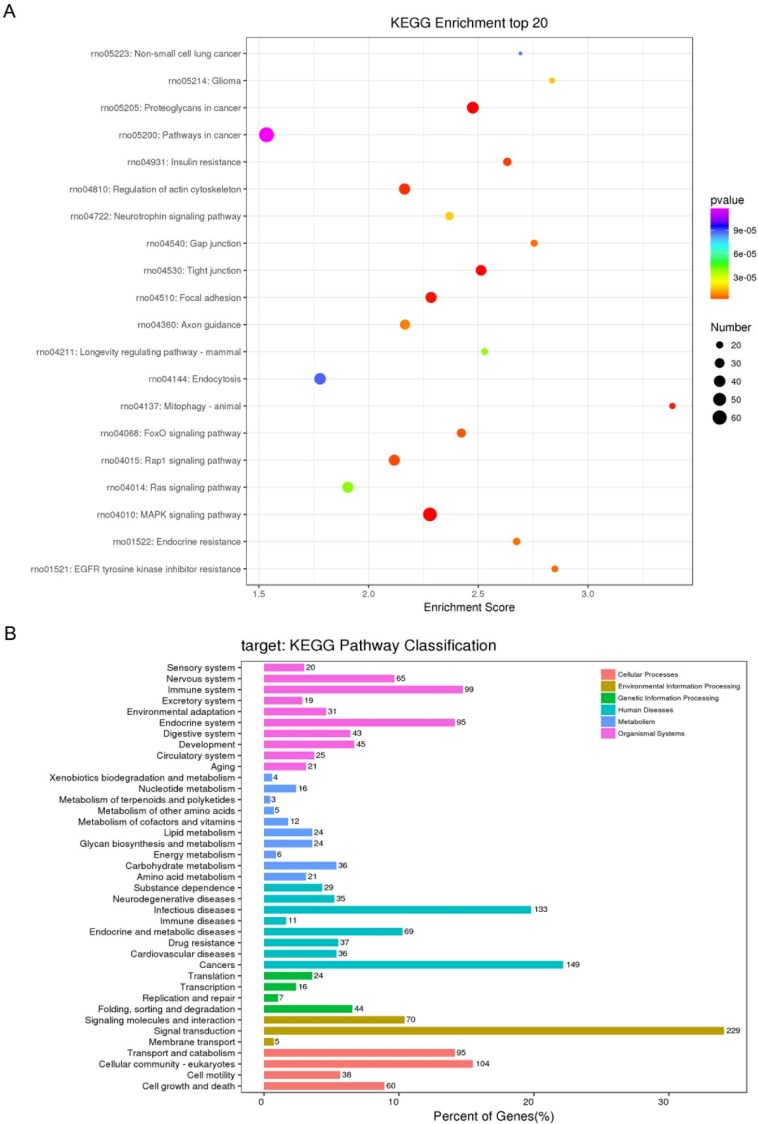
Kyoto Encyclopedia of Genes and Genomes (KEGG) pathway analysis of differentially expressed micro ribonucleic acids (miRNAs) (A) KEGG pathways of top20 enrichment score. (B) Distribution of KEGG Level2 of different miRNAs.

A KEGG pathway dot plot was constructed to show the significant enrichment pathway with the top 20 enrichment score values. KEGG pathway analysis predicted the pathways affected by the variation of miRNAs in exosomes from the plasma after TBI (Figure 4A). The top 20 pathways included MAPK signaling pathway, Proteoglycans in cancer, Tight junction, Focal adhesion, Mitophagy - animal, Regulation of actin cytoskeleton, Insulin resistance, Rap1 signaling pathway, FoxO signaling pathway, EGFR tyrosine kinase inhibitor resistance, Gap junction, Endocrine resistance, Axon guidance, Glioma, Neurotrophin signaling pathway, Longevity regulating pathway - mammal, Ras signaling pathway, Non-small cell lung cancer, Endocytosis, Pathways in cancer. Of all of these pathways, Mitophagy - animal seemed to have the highest enrichment score.

Similar to GO classification, KEGG classification counted the number or components of proteins or genes at the following function levels: cellular processes (transport and catabolism, cellular community-eukaryotes, cell motility, cell growth and death), environmental information processing (signaling molecules and interaction, signal transduction, membrane transport), genetic information processing (translation, transcription, replication and repair, fold, sorting and degradation), human diseases (substance dependence, neurodegenerative diseases, infectious diseases, immune diseases, endocrine and metabolic diseases, drug resistance, cardiovascular diseases, cancers), metabolism (Xenobiotics biodegradation and metabolism, nucleotide metabolism, metabolism of terpenoids and polyketides, metabolism of other amino acids, metabolism of cofactors and vitamins, lipid metabolism, glycan biosynthesis and metabolism, energy metabolism, carbohydrate metabolism, biosynthesis of other secondary metabolites, amino acid metabolism), and organismal systems (sensory system, nervous system, immune system, excretory system environmental adaptation, endocrine system, digestive system, development, circulatory system, aging) (Figure 4B).

**Figure 5:**
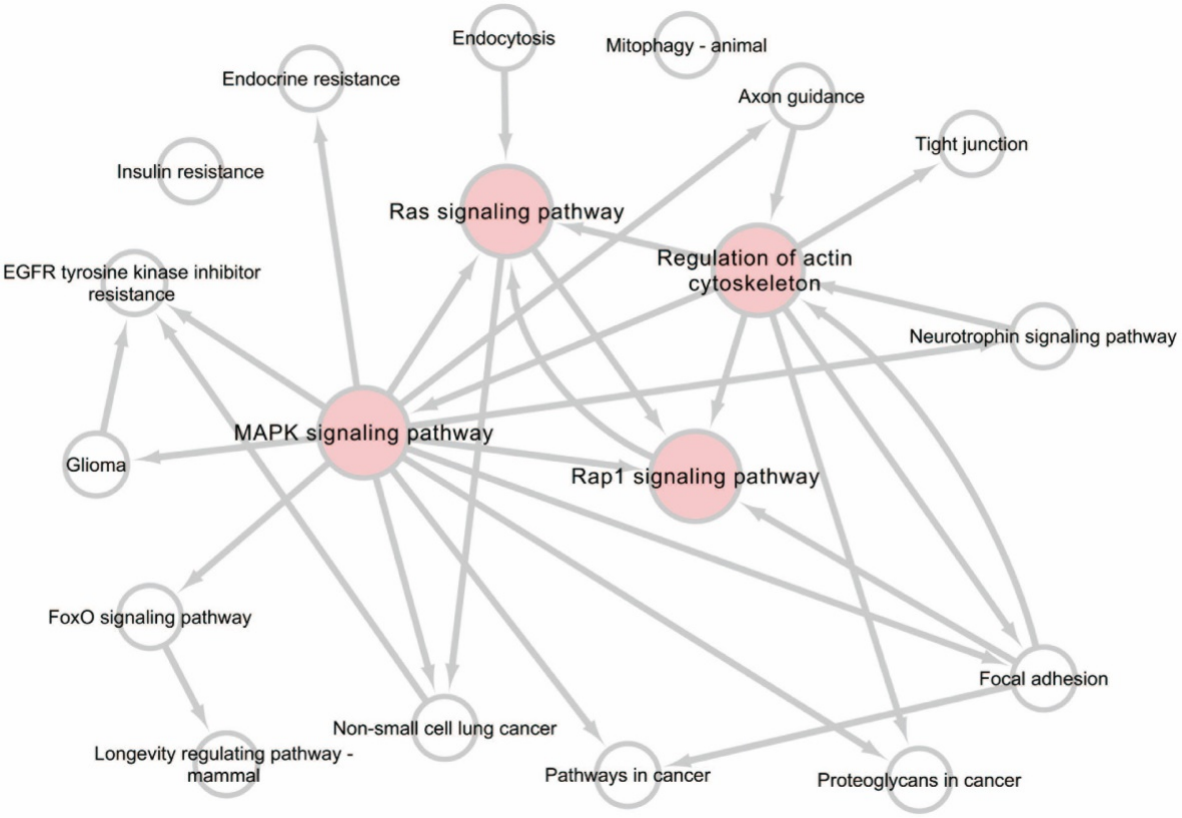
Pathway relation network analysis of the top 20 enrichment of miRNAs. The network was established based on the results of Kyoto Encyclopedia of Genes and Genomes (KEGG) pathway analysis and KEGG database search.

A pathway relationship network of the top 20 significant pathways of differentially expressed miRNAs was constructed (Figure 5), showing that the MAPK signaling pathway, Regulation of actin cytoskeleton, Rap1 signaling pathway, and Ras signaling pathway may have a regulatory effect on upstream levels.

### Validation of the accuracy of miRNA-seq data by qRT-PCR

**Figure 6:**
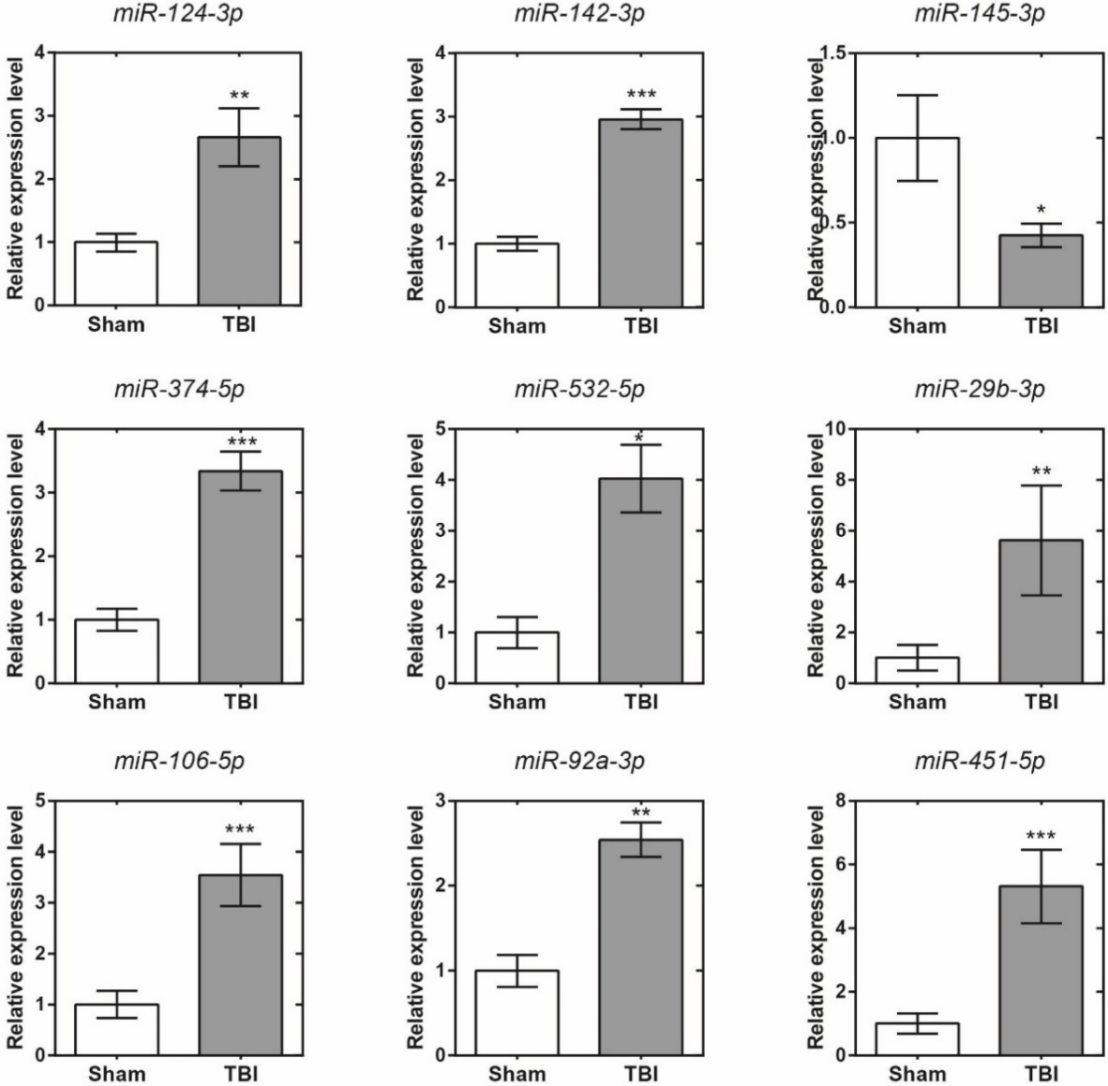
Quantitative reverse transcription polymerase chain reaction validation results of 9 selected micro ribonucleic acids. *, p<0.05; **, p<0.01, ***, p<0.001.

Eleven differentially expressed miRNAs were randomly selected for validation. The target gene expression levels of these RNAs were normalized to their internal control, U6. Comparisons of the expression levels of these miRNAs by sequencing and qRT-PCR are shown in Figure 6. The expression levels of these miRNAs were calculated using the 2^-δδCT^ method. The data are expressed as the means ± SD. There were significant differences in nine of all the selected miRNAs (*p*<0.05). Among them, the variation tendencies of miR-124-3p, miR-142-3p, miR-145-3p, miR-374-5p, miR-532-5p, miR-29b-3p and miR-106b-5p were in accordance with the micro sequencing; however, the variation tendencies of miR-92a-3p and miR-451-5p were not in accordance with the sequencing results. Moreover, although no significant differences were found (*p* > 0.05), the other miRNAs (miR-181c-3p and miR-9a-3p) exhibited concordant results with the miRNA sequencing results (data not shown). In total, the objective miRNA validation rate was 7/9, showing that the miRNA expression profiles were reliable.

## Discussion

The altered expression profile of miRNAs in exosomes from the plasma of peripheral blood after TBI in rats was studied for the first time by high-throughput whole transcriptome sequencing and subsequent bioinformatics analysis. We made several novel observations that improve the understanding of post-TBI molecular and intermolecular interactions as well as the key signaling pathways in which they participate. First, we demonstrated that the components of exosomes from plasma change after TBI. Exosomes were isolated and then confirmed by TEM, NTA and WES. The change in biological material in exosomes from plasma can reflect the physiological and pathological processes after TBI and may be a potential target of TBI therapy.

In this study, we found a total of 50 miRNAs in exosomes that were differentially expressed after TBI, including 31 upregulated and 19 downregulated miRNAs. These altered miRNAs might participate in the progression of TBI. Several previous studies have demonstrated the involvement of various spectrums of miRNAs from different sources in the TBI process, including miRNAs from cerebrospinal fluid (CSF) 22, serum 22, brain tissue 23, and saliva 32. Compared to these samples, CSF or brain tissue is difficult to obtain in an emergency room (ER), and preparation of serum usually takes hours, while plasma can be acquired relatively easily along with blood tests in ER. In the present study, miRNA samples were acquired from exosomes in plasma. We found a different spectrum of miRNAs compared to those found in previous studies, but this difference coincided with a previous study 33, which found that measurement of exosome miRNAs cannot be replaced by measurement of plasma miRNAs and vice versa. We suggest that this discrepancy may be due to sample disparity, miRNAs in plasma, plasma-exosomes, or the fact that other biofluids might be regulated by different mechanisms.

We found some miRNAs that were reported in previous studies. For example, miR124-3p has long been studied and reported to have a role in regulating hepatocellular carcinoma 34, breast cancer 35, 36, acute lymphoblastic leukaemia 37 and non-small cell lung cancer 38. It has also been reported in CNS diseases such as Alzheimer's disease 39 and glioma 40, 41. Previous studies 42 have shown that miR-124-3p is upregulated after TBI in brain tissue in microglial exosomes from the acute to the chronic phase of TBI, and increases in miR-124-3p in microglia promote an anti-inflammatory response in both *in vitro* and *in vivo* experiments. MiR-124-3p may also be a chronic regulator of gene expression after brain injury 43, therefore, miR-124-3p is a promising therapeutic target for interventions in neuroinflammation after TBI. Additionally, miR-142-3p, which has also long been studied, has been reported in several tumours 44-46 and other diseases 47, 48. Its expression in rat hippocampus after TBI has been reported 49, indicating that it may be a sensitive and informative biomarker for forensic assessment of TBI. As to miR-106-5p and miR-532-5p, they have been reported in ischemic stroke as biomarkers, and they may serve a novel role in the pathogenesis 50. Furthermore, miR-181c-3p has been shown in previous studies to be regulated by several circRNAs after TBI in mice, including chr17:39846653-39847022+, chr17:39846695-39847059+, and chr17:39846692-39847021+ 51. Although involvement of this mechanism has not been reported in TBI, it has been reported in other fields 52-54, indicating that it may have a therapeutic role in TBI treatment or assessment.

Moreover, to gain insight into the potential function of the differentially expressed miRNAs, KEGG pathway analysis was performed, which revealed that the differentially expressed miRNAs participated in several biological pathways. Of these pathways, the MAPK signaling pathway, Ras signaling pathway, and Rap1 signaling pathway are related to environmental information processing and signal transduction. Based on pathway analysis, we constructed a pathway relationship network, which indicated that the most important upstream pathway was MAPK signaling pathway and Ras signaling pathway, and Rap1 signaling pathway were downstream pathways also involved in this network. Many studies have indicated the role and the potential therapy of activation of RAS or RAP1 in traumatic brain injury 55-57. There were also several studies about the actin cytoskeleton, which showed the mechanism in TBI procedure 58, 59. As the upstream pathway, MAPK signaling pathway may serve as another important role.

The MAPK signaling pathway, which is a group of serine/threonine protein kinases involved in the cellular signal transduction 60, which regulated many cellular functions, affects a large number of human diseases. Previous studies have demonstrated the effect of MAPK signals on tumor metastasis 61, 62, osteogenesis 63, 64, osteoarthritis 65, 66, or certain inflammatory diseases 67. For example, it can regulate the proliferation and differentiation of brain cells 68. Furthermore, this pathway was also found to be involved in neuroinflammation in traumatic brain injury; according to a study that showed that p38α/MAPK regulates microglial to TBI mice 69. In other diseases, a research on osteoarthritis showed a suppressed miR-92-3p expression up activation of MAPK in chondrocytes 70; miR-221-5p regulates MAPK/ERK signaling pathway in prostate cancer cells, which suggested miR-221-5p plays an important role in prostate cancer progression 71; a research in glioma showed the upregulation of miR-124-3p induced cellular apoptosis via MAPK activation, and promote cell autophagy 72. These combined evidences showed several miRNAs in our studies are according with MAPK signaling pathway.

Last, we predicted the miRNAs that target the mRNA network according to the database and bioinformatics criteria. Briefly, the network showed that rno-miR-181c-3p, rno-miR-28-3p and rno-miR-451-5p regulated few mRNAs. In contrast, rno-miR-124-3p, rno-miR-106b-5p, rno-miR-29b-3p, and rno-miR-9a-5p, regulated a large number of mRNAs and seemed to be more important. According to our network and previous studies, these mRNAs and the related mRNAs may merit further research in the TBI process.

## Conclusion

In the present study, for the first time, we identified a series of differentially expressed miRNAs in exosomes obtained from the plasma after WD-induced TBI in rats, which may be related to the physiological and pathological processes that occur after TBI. We also predicted the potential roles of pathways and the interactions of miRNAs with mRNAs. The present study demonstrated that miRNA in plasma exosomes are altered after TBI, which suggests that they may serve as biomarkers for the diagnosis or merit consideration as therapeutic targets in treating TBI.

## Figures and Tables

**Table 1:** Read statistics.

Sample	Raw reads	Clean reads	miRNA nunber
*Sham1*	27,697,417	17,101,961	358
*Sham2*	38,841,725	27,519,720	367
*Sham3*	39,139,836	23,860,112	401
*TBI1*	27,397,880	16,609,560	370
*TBI2*	35,128,271	19,671,404	411
*TBI3*	48,542,524	26,323,056	361

miRNA, micro ribonucleic acid; TBI, traumatic brain injury.

**Table 2:** Top 50 of differentially expressed miRNAs.

miRNA ID	Log_2_FC	*p*-value	up/down	Sequence	Length
novel127_mature	-7.96	1.63E-04	Down	AGAAGAGGGAGCTGCAGCC	19
novel147_mature	8.16	2.76E-02	Up	GGAAGAGCAGCTGAGGCC	18
novel148_mature	9.82	3.51E-04	Up	CCATCTGGTAGCTGGTTCCT	20
novel150_mature	4.58	1.56E-02	Up	GGGGCGCGGGCGGGGCCGG	19
novel153_mature	8.88	3.78E-05	Up	AGACCTAGGCACTCAGAT	18
novel186_mature	8.50	1.18E-02	Up	GGAGGTGACTGCAGTGGTGC	20
novel214_mature	-1.59	9.26E-04	Down	CGGGGTACTGTAAGTGGC	18
novel217_mature	-7.36	1.03E-02	Down	GGCTGTCGGCGGTCTGCCA	19
novel239_mature	8.40	1.36E-02	Up	GGGAGCAGTAGCCTTGGGC	19
novel243_mature	8.24	1.45E-02	Up	GGCAGCTGAGGAAAGGGAC	19
novel260_mature	8.82	3.88E-03	Up	GGAGGGCTGGGCCTGGAC	18
novel274_mature	8.22	1.82E-02	Up	GAAGGGCTGGGAGGGTTGCC	20
novel283_mature	8.88	3.61E-03	Up	GGTGAGGTGGATGAGTGGG	19
novel294_mature	-7.39	3.18E-02	Down	AGAGGCAGAACAGGGTTACC	20
novel331_mature	8.94	2.94E-03	Up	GGTGTGTGGATGGGTAGGG	19
novel336_mature	-7.23	4.48E-02	Down	CCCTGCTGTCCCTGGGCC	18
novel341_mature	8.14	1.85E-02	Up	GGAGGTGGAGGAACGGCC	18
novel344_mature	7.71	4.44E-02	Up	GTTTGTGTGAGGGTTGTG	18
novel346_mature	2.11	3.19E-02	Up	CGGCCATGATGACACTCC	18
novel350_mature	-7.93	1.10E-02	Down	GGGGTCCTGGGTCTCAGCC	19
novel35_mature	7.99	2.09E-03	Up	AGGGAGCCCCGGCTGGTGGACGCC	24
novel360_mature	-7.40	2.95E-02	Down	GGAGGAATGTGAAGAGCC	18
novel366_mature	-1.26	4.83E-03	Down	AGGGTGTGTAGTGGAACC	18
novel39_mature	-2.53	9.16E-05	Down	GGAGAGACCACCCTAGAA	18
novel54_mature	7.15	2.28E-02	Up	GAAAGGGAAGCGCTTGTGT	19
novel57_mature	8.28	1.89E-03	Up	GAGGAACTCCGCCGCCTGGCGCC	23
novel65_mature	8.60	9.39E-04	Up	AGTCAGAACCTGAACGGCC	19
novel67_mature	-8.26	6.38E-03	Down	AGACATTGGACATCCGGGGC	20
novel88_mature	7.97	2.86E-02	Up	AGAGCTGGGGCACACAAG	18
novel98_mature	-7.35	2.96E-02	Down	AGTCCTGGCAGTGGCTCCC	19
novel99_mature	-7.47	2.80E-02	Down	GGAGAGGCAGCAGAGGGGC	19
rno-miR-106b-5p	4.45	1.03E-02	Up	TAAAGTGCTGACAGTGCAGAT	21
rno-miR-124-3p	2.31	2.12E-02	Up	TAAGGCACGCGGTGAATGCC	20
rno-miR-142-3p	7.58	1.82E-02	Up	TGTAGTGTTTCCTACTTTATGGA	23
rno-miR-145-3p	-6.86	1.02E-03	Down	GGATTCCTGGAAATACTGTTC	21
rno-miR-181c-3p	6.48	1.85E-02	Up	ACCATCGACCGTTGAGTGGACC	22
rno-miR-195-3p	7.88	3.47E-02	Up	CCAATATTGGCTGTGCTGCTCCA	23
rno-miR-221-5p	-2.68	2.23E-02	Down	ACCTGGCATACAATGTAGATTTC	23
rno-miR-28-3p	-2.32	2.74E-02	Down	CACTAGATTGTGAGCTCCTGGA	22
rno-miR-29b-3p	5.76	3.02E-03	Up	TAGCACCATTTGAAATCAGTGTT	23
rno-miR-328a-5p	7.67	4.98E-02	Up	GGGGGGCAGGAGGGGCTCA	19
rno-miR-361-3p	6.57	2.01E-02	Up	CCCCCAGGTGTGATTCTGATTCGT	24
rno-miR-374-5p	2.54	4.30E-02	Up	ATATAATACAACCTGCTAAGTG	22
rno-miR-434-3p	3.01	4.70E-02	Up	TTTGAACCATCACTCGACTCCT	22
rno-miR-451-5p	-1.26	2.03E-03	Down	AAACCGTTACCATTACTGAGTT	22
rno-miR-532-5p	8.48	8.87E-03	Up	CATGCCTTGAGTGTAGGACTGT	22
rno-miR-92a-3p	-1.04	8.06E-03	Down	TATTGCACTTGTCCCGGCCTG	21
rno-miR-96-5p	-2.54	2.91E-02	Down	TTTGGCACTAGCACATTTTTGCT	23
rno-miR-9a-3p	4.75	1.11E-02	Up	ATAAAGCTAGATAACCGAAAGT	22
rno-miR-9a-5p	-4.77	4.32E-02	Down	TCTTTGGTTATCTAGCTGTATGA	23

FC, fold change.

**Table 3:** Primers sequences used in this study.

miRNA	Primer type	Primer Sequence (5'-3')
rno-miR-124-3p	Forward	TAAGGCACGCGGTGAATGCC
rno-miR-142-3p	Forward	TGTAGTGTTTCCTACTTTATGGA
rno-miR-145-3p	Forward	GGATTCCTGGAAATACTGTTC
rno-miR-374-5p	Forward	ATATAATACAACCTGCTAAGTG
rno-miR-532-5p	Forward	CATGCCTTGAGTGTAGGACTGT
rno-miR-29b-3p	Forward	TAGCACCATTTGAAATCAGTGTT
rno-miR-106b-5p	Forward	TAAAGTGCTGACAGTGCAGAT
rno-miR-92a-3p	Forward	TATTGCACTTGTCCCGGCCTG
rno-miR-451-5p	Forward	AAACCGTTACCATTACTGAGTT

The reverse sequence and U6 sequence were provided in Mir-X miRNA First Strand Synthesis Kit (638313, TaKaRa, Osaka, Japan) as identified by previous studies.

## Abbreviations

TBI: Traumatic Brain Injury
CNS: Central Nervous System
miRNA: Micro Ribonucleic Acid
CD: Cluster of Differentiation
HSP: Hat Sock Protein
GO: Gene Oncology
KEGG: Kyoto Encyclopedia of Gene and Genomes
BP: Biological Process
CC: Cellular Component
MF: Molecular Function
